# Metformin Protects H9C2 Cardiomyocytes from High-Glucose and Hypoxia/Reoxygenation Injury via Inhibition of Reactive Oxygen Species Generation and Inflammatory Responses: Role of AMPK and JNK

**DOI:** 10.1155/2016/2961954

**Published:** 2016-05-16

**Authors:** Mingyan Hu, Ping Ye, Hua Liao, Manhua Chen, Feiyan Yang

**Affiliations:** Department of Cardiology, Central Hospital of Wuhan, Tongji Medical College, Huazhong University of Science and Technology, Wuhan 430014, China

## Abstract

Metformin is a first-line drug for the management of type 2 diabetes. Recent studies suggested cardioprotective effects of metformin against ischemia/reperfusion injury. However, it remains elusive whether metformin provides direct protection against hypoxia/reoxygenation (H/R) injury in cardiomyocytes under normal or hyperglycemic conditions. This study in H9C2 rat cardiomyoblasts was designed to determine cell viability under H/R and high-glucose (HG, 33 mM) conditions and the effects of cotreatment with various concentrations of metformin (0, 1, 5, and 10 mM). We further elucidated molecular mechanisms underlying metformin-induced cytoprotection, especially the possible involvement of AMP-activated protein kinase (AMPK) and Jun NH(2)-terminal kinase (JNK). Results indicated that 5 mM metformin improved cell viability, mitochondrial integrity, and respiratory chain activity under HG and/or H/R (*P* < 0.05). The beneficial effects were associated with reduced levels of reactive oxygen species generation and proinflammatory cytokines (TNF-*α*, IL-1*α*, and IL-6) (*P* < 0.05). Metformin enhanced phosphorylation level of AMPK and suppressed HG + H/R induced JNK activation. Inhibitor of AMPK (compound C) or activator of JNK (anisomycin) abolished the cytoprotective effects of metformin. In conclusion, our study demonstrated for the first time that metformin possessed direct cytoprotective effects against HG and H/R injury in cardiac cells via signaling mechanisms involving activation of AMPK and concomitant inhibition of JNK.

## 1. Introduction

Diabetes mellitus is associated with a number of long-term complications, including nephropathy, retinopathy, stroke, and cardiovascular diseases, which lead to decreased quality of life and reduced life expectancy [1]. Patients with type 2 diabetes mellitus (T2DM) have a higher risk for coronary heart disease [2] and are more susceptible to myocardial ischemia/reperfusion (I/R) injury as compared with nondiabetic individuals [3, 4]. To date, no agent is in routine clinical use to protect the myocardium against I/R injury, although several pharmacological agents have been studied with respect to their ability to attenuate I/R injury [5]. Metformin (1,1-dimethylbiguanide), a biguanide derivate, is the most widely prescribed drug in the treatment of T2DM [6]. Clinical trials demonstrated that metformin reduced diabetes-related death and all-cause mortality [7, 8] and previous exploratory studies suggested that metformin had direct vascular beneficial effects, for example, in a murine model of myocardial I/R, but the underlying mechanisms of this beneficial effect are not completely understood [9, 10].

The pathogenesis of hypoxia/reoxygenation (H/R) injury (a major component of I/R injury) in diabetic hearts is associated with cardiomyocyte apoptosis [11] and overproduction of reactive oxygen species (ROS) [12]. It is widely accepted that metformin leads to activation of AMP-activated protein kinase (AMPK) with increased levels of phosphorylated AMPK [13, 14], which has complex properties on cardiomyocyte functionality and ROS production [12]. In this context, we hypothesized that metformin played a direct protective role against I/R injury in diabetic hearts and we tested this hypothesis in an in vitro study using H9C2 rat cardiomyoblasts exposed to H/R injury under a simulated hyperglycemic (HG) condition with or without coincubation with various concentrations of metformin. We further investigated the potential cellular and molecular mechanisms underlying metformin-induced cytoprotection against HG and/or H/R injury, particularly those related to the AMPK and JNK related kinase signaling pathways. Cellular ROS generation and proinflammatory cytokines were also investigated.

## 2. Methods

### 2.1. Cell Culture and Treatment Protocol

H9C2 rat cardiomyoblast cell line was purchased from the American Type Culture Collection (ATCC) and cultured in mixed growth medium (Dulbecco's modified Eagle's medium (DMEM) (Hyclone)) supplemented with 10% heat-inactivated FBS (Hyclone). Cells were kept in an incubator in an atmosphere of 5% CO_2_ and 95% air at 37°C and passaged at 1 : 3 ratio when they reached 80% confluence. For the H/R experimental groups, cells were firstly maintained at 37°C under hypoxic atmosphere of 95% N_2_ and 5% CO_2_ for 3 hours, and then cells were given fresh medium with serum and maintained in normoxic conditions (i.e., reoxygenation) for the next 3 hours. The oxygen content (~1% O_2_) inside the incubator was continuously monitored to maintain a stable level of hypoxia. In the HG (33 mM)- and metformin (0, 1, 5, and 10 mM)-treated groups, the cells were pretreated with glucose for 48 hours with or without an inhibitor of AMPK (compound C (Tocris)) or an activator of JNK (anisomycin (ANISO, Sigma-Aldrich)) followed by preincubation for 30 min before addition of metformin, which was added 60 min prior to H/R until the end of the experiment protocol. JNK inhibitor (SP600125, Sigma-Aldrich) was added 30 min ahead of H/R. The cells in the control group were treated with the same procedure under normal culture conditions.

### 2.2. Cell Viability

After exposure to the abovementioned HG and/or H/R treatments with or without various concentrations of metformin (0, 1, 5, and 10 mM) and other kinase activators/inhibitors, cell proliferation in the H9C2 cells was assessed using Cell Counting Kit-8 (CCK-8, Sigma-Aldrich) according to the manufacturer's instruction. Briefly, cells were seeded on 96-well plates at a density of 2 × 10^3^ per well in 100 *μ*L of complete medium. After being treated with the corresponding drug(s) and H/R as described above, the cells were further incubated with 10 *μ*L of the CCK-8 reagent for 0.5 to 4 hours. The absorbance at 450 nm wavelength was measured with a microplate reader (BIO-TEX ELx800).

### 2.3. Measurement of Mitochondrial Membrane Potential

As a mitochondria specific membrane potential-sensitive fluorescent probe, JC-1 (Keygen) accumulates in the mitochondrial matrix when mitochondrial membrane potential is high, forming J-aggregates which emit bright yellow-red fluorescence. In contrast, JC-1 presents itself as a green fluorescent monomer with low levels of aggregation when mitochondrial membrane potential is low. During this assay, cells were seeded on 6-well plates and, following the assigned drug and/or H/R treatments, the cells were loaded with JC-1 for 20 min at 37°C and then harvested to detect fluorescence with a flow cytometer (BD Biosciences).

### 2.4. Measurement of Activities of Mitochondrial Complex I and Complex III

Mitochondrial complex activities were measured by a commercial assay kit for mitochondrial complex activity (Beyotime Institute of Biotechnology, China) following the manufacturer's instruction. Briefly, NADH-cytochrome c reductase activity (complexes I–III) was measured at 550 nm in a reaction medium containing (mM) 100 phosphate buffer (pH 7.4), 0.2 NADH, 0.1 cytochrome c, and 0.5 KCN at 30°C. Enzyme activity was expressed in nmol cytochrome c reduced per minute per mg of protein. The activity was measured by the absorbance at 340 nm wavelength with a microplate reader (BIO-TEX ELx800) for 3 min.

### 2.5. Flow Cytometric Evaluation of Intracellular ROS

The ROS generation was monitored by flow cytometry using peroxide-sensitive fluorescent probe 2,7-dichlorofluorescein diacetate (DCFH-DA) as previously described [15]. The cells were loaded with 10 mM DCFH-DA in serum-free medium at 37°C for 30 mins, then washed twice with PBS, and then monitored with a flow cytometer (BD Biosciences) at an excitation wavelength of 488 nm and an emission wavelength of 525 nm. ROS was determined by comparing the changes in fluorescence intensity to those of the control wells. The vertical coordinate represents the amount of cells, and the horizontal ordinate represents the mean fluorescence. The more the curve shifts to the right, the stronger the mean fluorescence is.

### 2.6. Real-Time PCR (Polymerase Chain Reaction) and mRNA Levels of Cytokines

Total RNA from the cultured cells was extracted with TRIzol reagent (Invitrogen, Carlsbad, CA), followed by the synthesis of first-strand cDNA (Thermo). Real-time quantitative PCRs were performed using the SYBR-green I Core Kit (Thermo). PCR products were detected in the ABI PRISM 7700 sequence detection system (Applied Biosystems), and the results were analyzed using the 2^−ΔΔCT^ method. The level of expression of mRNA was normalized to mRNA of GAPDH. Sequences of the primers are shown below:

 
*TNF-α:*
 F5′-GGTCTGAGTACATCAACCTGGA-3′,
 R5′-GGTCTGAGTACATCAACCTGGA-3′.
 
*IL-1α:*
 F5′-AAGACAAGCCTGTGTTGCTGAAGG-3′,
 R5′-TCCCAGAAGAAAATGAGGTCGGTC-3′.
 
*IL-6:*
 F5′-TCAAGGGAAAAGAACCAGACA-3′,
 R5′-TCAAGGGAAAAGAACCAGACA-3′.
 
*GAPDH:*
 F5′-CTCTCTGCTCCTCCCTGTTC-3′,
 R′5′-GCCAAATCCGTTCACACCG-3′.

### 2.7. Western Blotting

Western blotting was performed as previously described [15]. Briefly, equal amount of protein extraction was loaded into an electrophoresis apparatus and, after the completion of electrophoresis, the blot was transferred onto a PVDF membrane followed by timed incubation with the selected primary and secondary antibodies. The scanned images of Western blot bands were analyzed using the Quantity One software (Bio-Rad). The levels of protein expression were quantified by densitometry and normalized to *β*-actin expression. Primary antibodies including phosphorylated and total AMPK (P-AMPK/T-AMPK) and phosphorylated and total ACC (P-ACC/T-ACC) were purchased from Abcam, and phosphorylated and total JNK (P-JNK/T-JNK) antibodies were from Cell Signaling Technology.

### 2.8. Statistical Analyses

Each of the cellular and molecular biology assays was replicated with 3 independent experiments and the flow cytometry studies were repeated 6 times. All data were presented as mean ± standard deviation (SD). Comparisons between groups were performed by one-way ANOVA with Student-Newman-Keuls post hoc analyses. The level for significant statistical differences was set at *P* < 0.05.

## 3. Results

### 3.1. Metformin Protected against HG and H/R Induced Cardiac Cell Injury

**Figure 1:**
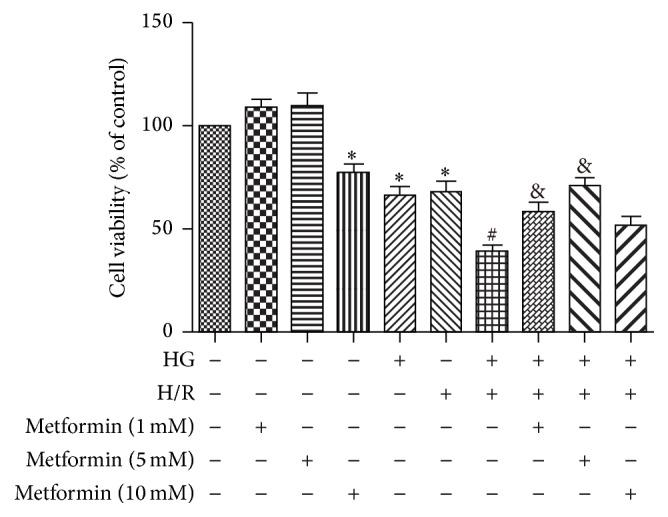
Dose-dependent effects of metformin on HG + H/R induced reduction in cell viability. H9C2 cells were treated with different concentrations of metformin (0, 1, 5, and 10 mM). A “+” symbol indicates presence and a “−” symbol indicates absence of the relevant treatment condition, such as HG (33 mM), H/R, and various concentrations of metformin. Cell viability of each group was estimated using the CCK-8 assay. Data are shown as means ± SD of 3 independent experiments. ^*∗*^ *P* < 0.05 versus Control; ^#^ *P* < 0.05 versus HG or H/R; ^&^ *P* < 0.05 versus metformin + HG + H/R.

To observe the effect of metformin on cell viability, H9C2 cells were exposed to HG and H/R treatments along with various concentrations of metformin. As evidenced by CCK-8 assay, the cell viability of HG or H/R groups was significantly lower than those of control group and the cells exposed to HG + H/R had greater loss in cell viability as compared with HG and H/R alone groups. Introducing low concentrations (1 or 5 mM) of metformin into the cell culture medium significantly increased the H9C2 cells viability while a higher concentration of metformin (10 mM) aggravated the loss in cell viability induced by HG + H/R treatment (Figure 1), indicating dose-dependence for metformin-induced protective effects, and only a moderate concentration of metformin is cytoprotective against HG + H/R injury.

### 3.2. Dependence of AMPK Activation in Protection of Metformin against H/R Injury

**Figure 2:**
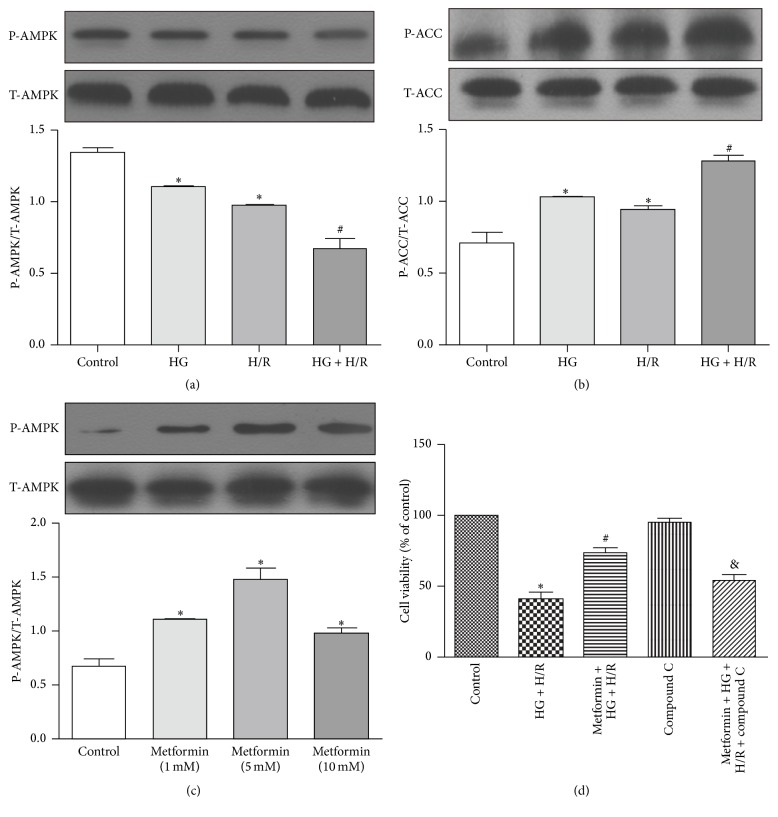
AMPK pathway was inhibited during H/R injury and exacerbated by HG and the cytoprotective effects of metformin were dependent on AMPK activation. H9C2 cells were treated with HG (33 mM), H/R, and HG + H/R. The expression of P-AMPK, T-AMPK (a) and P-ACC, T-ACC (b) was measured by Western blots. (c) Expression of P-AMPK and T-AMPK in the H9C2 cells treated with different concentrations of metformin (0, 1, 5, and 10 mM) was measured by Western blots. (d) Cells were treated with HG + H/R, metformin (5 mM) + HG + H/R, compound C (1 *μ*M), or metformin + HG + H/R + compound C. Cell viability of each group was established using the CCK-8 assay. ^*∗*^ *P* < 0.05 versus control; ^#^ *P* < 0.05 versus HG or H/R; ^&^ *P* < 0.05 versus metformin + HG + H/R. Data are shown as means ± SD of 3 independent experiments.

It was reported that metformin protected cardiomyocytes from injury through activation of AMPK pathway [13, 14]. To examine whether AMPK pathway inactivation was involved in HG and/or H/R induced cardiac cell injury, we tested the phosphorylation state of AMPK and its downstream target ACC. Our results showed that the levels of P-AMPK were markedly lower and its inhibitory downstream target P-ACC were higher in HG or H/R groups than in control group (Figures 2(a) and 2(b)) and the cells in HG + H/R group had the lowest level of P-AMPK (Figure 2(a)) and the resultant highest level of P-ACC (Figure 2(b)). These results suggested that AMPK pathway was inhibited during H/R injury and this effect was exacerbated under HG + H/R injury. Incubation with all three concentrations of metformin (1, 5, and 10 mM) significantly increased P-AMPK expression and the highest level of P-AMPK was found in the cytoprotective dose (5 mM) of metformin (Figure 2(c)). Furthermore, the metformin-enhanced cell viability under HG + H/R conditions was abolished by compound C, an inhibitor of AMPK (Figure 2(d)), confirming that metformin protected against H/R + HG injury by activating AMPK pathway.

### 3.3. Metformin Attenuates HG + H/R Induced Reduction in Mitochondrial Transmembrane Potential and Increased ROS Formation

**Figure 3:**
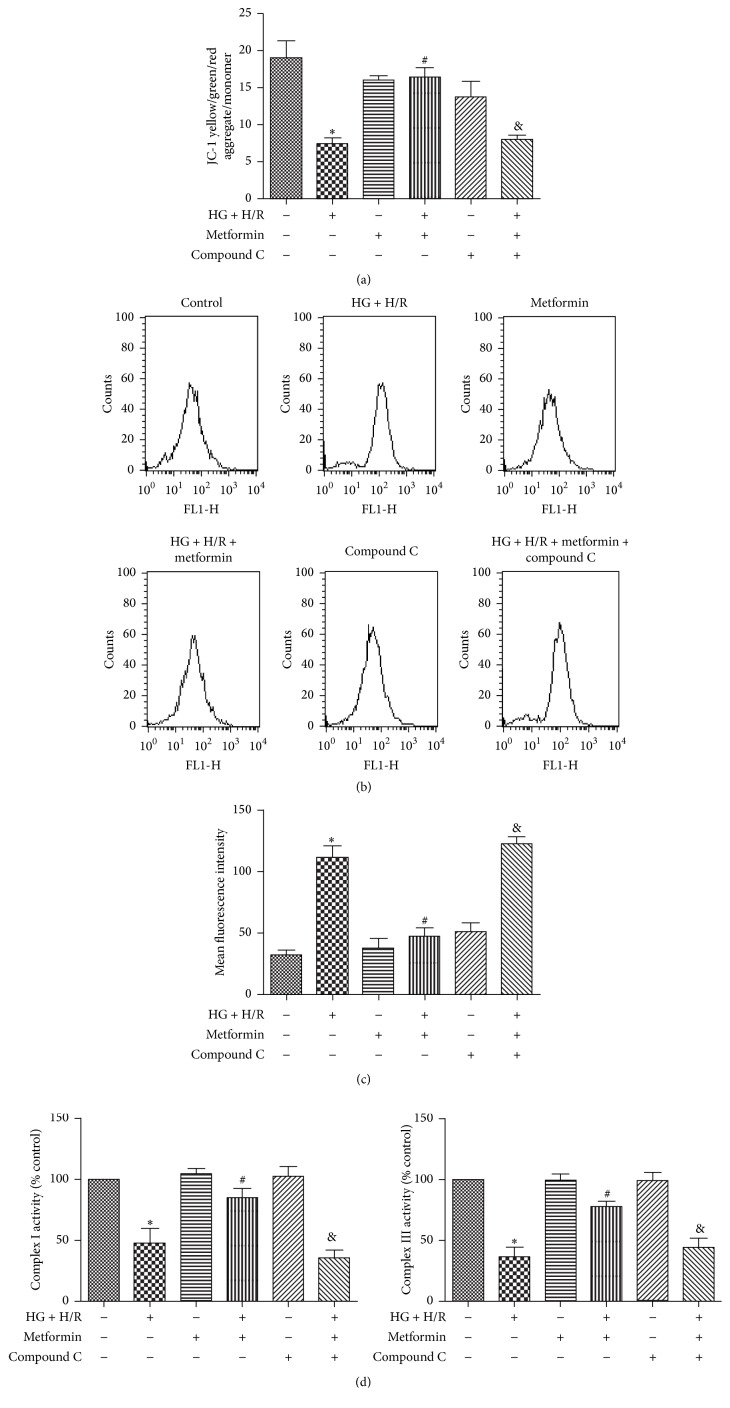
Metformin attenuated HG + H/R induced decrease in cell viability by preservation of mitochondrial membrane integrity and mitigating oxidative stress. H9C2 cells were treated with HG + H/R, metformin (5 mM), metformin + HG + H/R, compound C (1 *μ*M), and metformin + HG + H/R + compound C. (a) Mitochondrial membrane potential evidenced by JC-1 staining. (b) ROS generation was measured by the DCF fluorescence intensity. (c) Bars represent quantified ROS generation. (d) Mitochondrial electron transport chain complex I and complex III activities were measured with commercial kits. ^*∗*^ *P* < 0.05 versus control; ^#^ *P* < 0.05 versus HG + H/R; ^&^ *P* < 0.05 versus metformin + HG + H/R. Data in the bar graphs are calculated as means ± SD of 3 independent experiments.

Mitochondria act as a nexus for reperfusion injury pathways [16] and diffusion of mitochondrial transmembrane potential (Δ*ψm*) indicates mitochondrial dysfunction [17]. In our present study, a mitochondrial membrane potential kit was used to explore the effect of metformin on mitochondrial function in cells exposed to HG and H/R. Metformin ameliorated depolarized Δ*ψm* induced by HG + H/R treatment, which was abolished by compound C evidenced from the data of JC-1 (Figure 3(a)).

I/R injury impairs the mitochondrial respiratory chain, especially complexes I and III, and produces a large amount of ROS [18]. To investigate the effect of metformin on ROS levels in the cells exposed to HG and H/R, DCF-DA assay was performed. The levels of ROS in the HG + H/R groups treated with low dose metformin (5 mM) were significantly lower than that of the HG + H/R group (Figures 3(b)-3(c)). On the other hand, metformin increased the activity of mitochondrial electron transport chain complexes I and III that were reduced by HG + H/R (Figure 3(c)). Consistent with JC-1, the ROS reduction and mitochondrial electron transport chain complex activity increase by metformin treatment were reversed by compound C (Figures 3(b)–3(d)).

### 3.4. Metformin Inhibits Inflammatory Response to HG and H/R Injury

**Figure 4:**
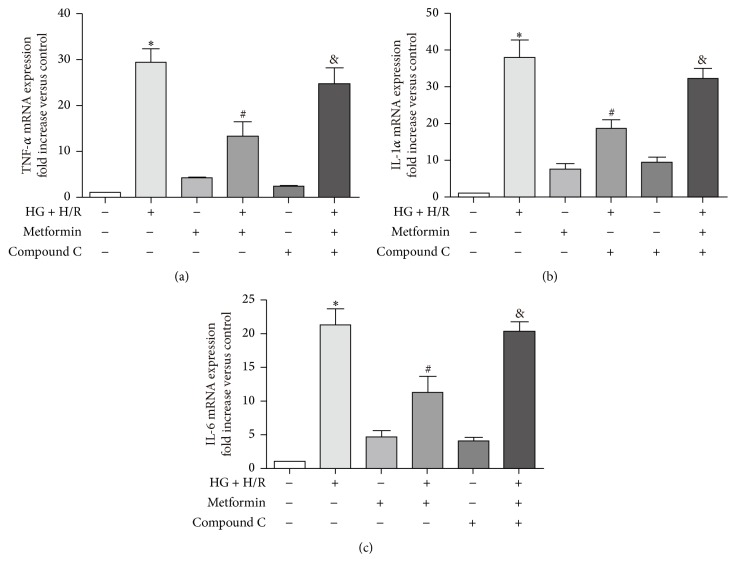
Metformin attenuated HG and H/R injury by inhibiting proinflammatory cytokine expression in cardiac cells. H9C2 cells were treated as described in Figure 3. (a)–(c) Expressions of TNF-*α*, IL-1*α*, and IL-6 mRNA level were measured by real-time PCR. GAPDH was used as the housekeeping gene. Data are shown as means ± SD of 3 independent experiments. ^*∗*^ *P* < 0.05 versus control; ^#^ *P* < 0.05 versus HG + H/R; ^&^ *P* < 0.05 versus metformin + HG + H/R.

Exposure to HG + H/R injury induced an inflammatory response, characterized by increasing mRNA levels of IL-1*α*, IL-6, and TNF-*α* compared with that of control groups (Figure 4) and administration of metformin (5 mM) reduced these changes. In addition, inhibition of AMPK by compound C reversed this trend, implying that the protective effect of metformin against HG + H/R induced inflammation was mediated by activation of AMPK signaling pathway.

### 3.5. Metformin Protects against HG + H/R Injury by Inhibiting Phosphorylation of JNK, Which Is a Downstream Target of AMPK

**Figure 5:**
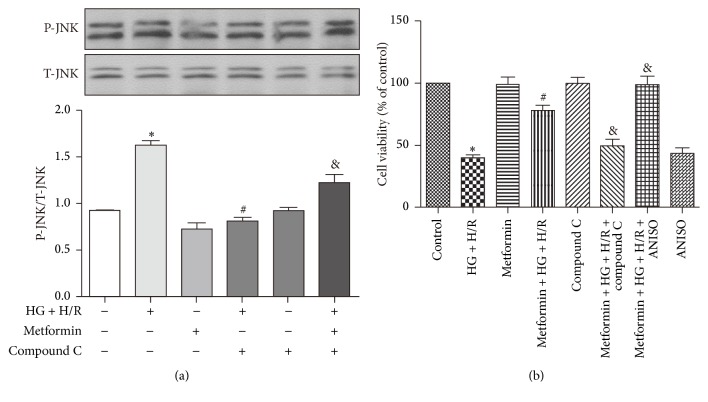
Metformin protects against HG + H/R induced cardiac cell injury by inhibiting JNK signaling pathway, which acts downstream of AMPK. H9C2 cells were treated with HG + H/R, metformin (5 mM), metformin + HG + H/R, compound C (1 *μ*M), metformin + HG + H/R + compound C, ANISO (10 *μ*M), and metformin + HG + H/R + ANISO. (a) Expression of P-JNK and T-JNK in each of the treatment conditions was measured by Western blots. (b) Effect of a putative JNK activator ANISO on cell viability was determined using the CCK-8 kit. Data are shown as means ± SD of 3 independent experiments. ^*∗*^ *P* < 0.05 versus control; ^#^ *P* < 0.05 versus HG + H/R; ^&^ *P* < 0.05 versus metformin + HG + H/R.

Expression of P-JNK and T-JNK in each of the treatment conditions was measured by Western blots (Figure 5(a)). The results showed that increased protein level of P-JNK during HG + H/R treatment was also decreased by metformin (5 mM) and inhibition of AMPK by compound C reversed these changes in P-JNK expression. In addition, the protective effect of metformin was abolished by cotreatment with a putative JNK activator, ANISO (Figure 5(b)), indicating that the inhibition of JNK is required for the metformin-induced cytoprotection.

### 3.6. JNK Inhibition Reduced HG and H/R Injury

**Figure 6:**
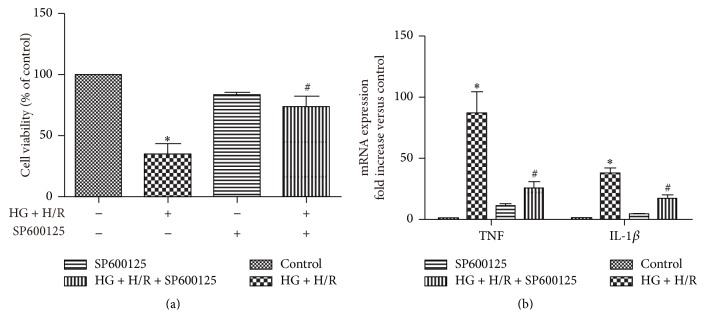
Protection of JNK inhibitor SP600125 against HG + H/R induced loss of cell viability and proinflammatory cytokines release. (a) Protective effects of SP600125 on HG + H/R induced loss of cell viability. (b) Effect of SP600125 on HG + H/R induced proinflammatory cytokine expression. Data are shown as mean ± SD of 3 independent experiments. ^*∗*^ *P* < 0.05 versus control; ^#^ *P* < 0.05 versus HG + H/R.

To further demonstrate that JNK signal pathway plays an essential role in HG and H/R induced cardiac injury, we performed additional experiments and found that a JNK inhibitor (SP600125) prevented the HG + H/R induced loss of cell viability and suppressed the inflammation cytokines synthesis under HG + H/R (Figure 6).

## 4. Discussion

The main finding of our study was that metformin protected cardiac cells against HG + H/R injury by a mechanism involving P-AMPK, ROS, and JNK signaling pathways. Patients with diabetes mellitus are at higher risk of cardiovascular events compared with nondiabetic individuals as evidenced by numerous clinical studies [19, 20]. For instance, diabetic patients are more vulnerable to ischemic heart diseases [3, 4]. Unfortunately, many therapeutic strategies that have been shown to be effective in the protection of nondiabetic hearts against I/R injury often lose their effectiveness in diabetic states [21, 22]. Although metformin has been reported to protect diabetic mouse heart against I/R injury [23, 24], its underlying mechanisms remain largely unknown. Our current study showed that metformin at a low concentration (5 mM) reduced the cardiac cell death caused by HG + H/R injury and metformin alone had no effect on cell viability. However, a higher concentration of metformin (10 mM) dramatically decreased cell viability. This cytotoxic effect of higher dose of metformin may result from the excessive enhancement of AMPK activity that led to suppression of platelet-derived growth factor receptor (PDGFR) as previously reported in H9C2 cells [25].

ROS plays a key role in HG + H/R induced cardiac injury and most of the ROS are generated within the impaired mitochondria. Metformin has been shown to reduce HG-induced ROS generation and oxidative stress in endothelial cells [26, 27]. Therefore, we investigated whether metformin had a suppressive effect against HG + H/R induced ROS overgeneration in cardiomyocytes. We first found that mitochondrial transmembrane potential (Δ*ψm*) was diffused after HG + H/R treatment indicating mitochondrial dysfunction and this effect was reversed by metformin (5 mM) coincubation (Figure 3(a)). In line with the result of mitochondrial function, metformin administration reduced the increase in ROS generation stimulated by HG + H/R (Figures 3(b)-3(c)). We subsequently examined whether the ROS was produced at the site of mitochondrion. As expected, the activity of mitochondrial electron transport chain complex I and complex III was reduced by HG + H/R injury and metformin attenuated this effect (Figure 3(d)). Overall, our data suggested that metformin protected heart cells against HG + H/R injury by inhibiting overproduction of ROS derived most likely from mitochondria.

Oxidative stress links multiple risk factors to disease and one of the main possible underlying mechanisms is that overproduction of ROS stimulates inflammatory response [28]. We found here that HG + H/R induced cardiac cell injury was associated with activation of inflammatory response as evidenced by the significant increase in mRNA of proinflammatory cytokines (TNF-*α*, IL-1*α*, and IL-6) and metformin mitigated these increases (Figures 4(a)–4(c)). Previous studies reported that exogenous ROS could stimulate JNK in H9C2 cells [29, 30]. Consistent with these previous studies, our present research also demonstrated that HG + H/R injury induced cardiac ROS overproduction was associated with an increase in P-JNK expression, which was inhibited by metformin administration (Figures 6(a)-6(b)).

Previous rat study reported that metformin activated AMPK pathway and protected against myocardial I/R injury [31]. To investigate whether metformin protected H9C2 cells against HG + H/R injury also through activation of AMPK, we used AMPK inhibitor compound C and found that the protective effect of metformin against HG + H/R injury was abolished by compound C treatment (Figure 5(b)). We further uncovered that the AMPK activation and cytoprotective effect of metformin were interconnected with inhibition on P-JNK and compound C restored the metformin-induced suppression of JNK, indicating that AMPK activation was an upstream event of JNK inhibition caused by metformin (Figures 6(a)-6(b)). Furthermore, the JNK activator anisomycin (ANISO) antagonized the inhibitory effects of metformin on HG + H/R induced JNK activation and also blocked cytoprotection of metformin (Figure 5(b)). Finally, our results provided additional evidence for a detrimental role played by JNK in HG + H/R injury, because a JNK inhibitor (SP600125) reduced the cell injury (Figure 6(a)) and proinflammatory cytokine response (Figures 6(a)-6(b)) caused by HG + H/R.

## 5. Conclusion

The current study has revealed for the first time that the in vitro direct treatment of metformin (5 mM) in H9C2 cardiomyoblasts attenuated HG and H/R induced cell injury, mitochondrial dysfunction, ROS overgeneration, and inflammatory response through an AMPK/JNK-dependent signaling pathway. These results from cultured cardiac cells may also implicate an important mechanism by which metformin antagonizes myocardial I/R injury in vivo. Future studies are needed to further validate the role of this signaling mechanism in mediating metformin-induced cardioprotection in various animal species including human.
